# A WeChat-Based Decision Aid Intervention to Promote Informed Decision-Making for Family Members Regarding the Genetic Testing of Patients With Colorectal Cancer: Randomized Controlled Trial

**DOI:** 10.2196/60681

**Published:** 2025-04-21

**Authors:** Huanhuan Li, Yanjie Zhao, Wei Li, Wenxia Wang, Shengze Zhi, Yifan Wu, Qiqing Zhong, Rui Wang, Jiao Sun

**Affiliations:** 1 School of Nursing Jilin University Changchun, Jilin China; 2 School of Nursing Anhui Medical University Hefei, Anhui China; 3 School of Nursing Xinjiang Medical University Urumqi, Xinjiang China; 4 Hebei General Hospital Shijiazhuang, Hebei China; 5 School of Nursing Peking University Beijing China

**Keywords:** decision aid, genetic testing, hereditary colorectal cancer, informed decision-making, RCT, WeChat based

## Abstract

**Background:**

Identifying patients with inherited colorectal cancer (CRC) syndromes offers many potential benefits. However, individuals often experience decisional conflict regarding genetic testing for CRC, and the uptake rate remains low. Given the growing popularity of genetic testing and the increasing demands on genetic service providers, strategies are needed to promote informed decision-making, increase genetic testing uptake among at-risk individuals, and ensure the rational use of genetic service resources.

**Objective:**

This study aims to determine whether a decision aid (DA) tool could promote informed decision-making among family members regarding the genetic testing of a patient with CRC.

**Methods:**

A single-center, parallel-group, randomized controlled trial was conducted. We randomized 82 family members of patients with CRC, who were involved in major medical decision-making for the patient, to either a DA intervention or usual care. The primary outcome was informed decision-making, assessed through measures of knowledge, decisional conflict, decision self-efficacy, and preparation for decision-making. Secondary outcomes included patients’ uptake of genetic counseling and testing, participants’ CRC screening behavior, healthy lifestyle scores, anxiety and depression levels, quality of life, and satisfaction with the intervention. Data were collected at baseline (T0), after the intervention (T1), and 3 months after the baseline survey (T2). The DA intervention and outcome assessments at T1 and T2 were delivered via WeChat. The effects of the intervention were analyzed using generalized estimating equation models.

**Results:**

Statistically significant improvements were observed in knowledge (T1: β=2.049, *P*<.001; T2: β=3.317, *P*<.001), decisional conflict (T1: β=–11.660, *P*<.001; T2: β=–17.587, *P*<.001), and decision self-efficacy (T1: β=15.353, *P*<.001; T2: β=22.337, *P*<.001) in the DA group compared with the usual care group at both T1 and T2. Additionally, the DA group showed significantly greater improvement in processed and red meat intake (β=–1.494, *P*<.001) at T1 and in healthy lifestyle scores (β=1.073, *P*=.03) at T2. No differences were found between the groups for other outcomes.

**Conclusions:**

A DA tool may be a safe, effective, and resource-efficient approach to facilitate informed decision-making about genetic testing. However, the current DA tool requires optimization and further evaluation—for example, by leveraging more advanced technology than WeChat to develop a simpler and more intelligent DA system.

**Trial Registration:**

Chinese Clinical Trial Registry ChiCTR2100048051; https://www.chictr.org.cn/showproj.html?proj=129054

## Introduction

Colorectal cancer (CRC) ranks third in incidence and second in mortality among all cancers worldwide [1]. Approximately 5%-10% of CRC cases are attributed to well-defined hereditary CRC syndromes, such as Lynch syndrome and familial adenomatous polyposis [2]. Individuals carrying mutated genes associated with hereditary CRC syndromes have a significantly higher risk of developing cancer [3]. The NCCN (National Comprehensive Cancer Network) Clinical Practice Guidelines for CRC Genetic/Familial High-Risk Assessment recommend that all individuals newly diagnosed with CRC undergo risk assessment for hereditary CRC syndromes through tumor-based microsatellite instability or immunohistochemistry testing, or through genetic counseling and patient education based on personal and family history, conducted by a qualified professional [3]. Further genetic testing using blood or saliva to diagnose hereditary CRC syndromes is recommended based on the results of the risk assessment [3]. A patient’s genetic testing results have important implications for the future uptake of genetic screening by other family members. Identifying inherited CRC syndromes not only provides an opportunity to optimize therapy and manage future risk for patients, but also offers risk-reduction strategies (eg, healthy lifestyle) and early-detection options for asymptomatic carriers of inherited CRC syndrome–associated mutations within the family [3,4].

Despite these benefits, cancer-related genetic screening remains in its infancy in many countries, including China. The implementation of universal tumor screening has been slow, and risk assessment and referral for hereditary CRC syndromes are not widely practiced [5-8]. Today, patients and their family members increasingly value information on the genetic causes of CRC and show growing interest in genetic testing [9,10]. However, the limited availability of genetic counseling services does not adequately meet the needs of patients and their families [11,12]. Patients with CRC or their family members in China have reported difficulty in accessing professional knowledge about hereditary CRC syndromes and related genetic testing [9,13]. A lack of knowledge serves as a barrier to informed decision-making regarding genetic testing [14,15]. The decision-making process itself is complex and challenging. Individuals must navigate various limitations of genetic testing, such as understanding its purpose and the implications of uncertain test results [16], as well as considering the potential impact on their psychosocial well-being [17], family relationships [18], and life insurance options for themselves and their offspring [19]. Individuals often experience decisional conflict regarding CRC genetic testing, and some patients and relatives undergo testing without receiving sufficient information, which may lead to postdecision regret [9,13] or inefficient use of genetic service resources. Given the complexity of the potential benefits and limitations of genetic testing for hereditary CRC, along with the unmet information needs of patients and families, the detection of hereditary CRC remains suboptimal [20-22]. Therefore, strategies are needed to promote informed decision-making, increase the uptake of genetic testing among at-risk individuals, and ensure the rational use of genetic service resources.

Studies in patients with CRC suggest that risk assessment, education, or decision aid (DA) interventions support informed decision-making and lead to higher test uptake among individuals at risk [16,23-26]. Compared with risk assessment and education alone, DAs are specifically designed to facilitate decision-making by enhancing individuals’ understanding of the potential benefits and risks of different options, and by helping them consider the personal importance they place on each option [27]. Recent reviews have shown that many DAs have been developed for decisions related to genetic testing for hereditary breast or ovarian cancer syndrome–associated mutations, and have confirmed the advantages of DAs in the decision-making process: mutation carriers who used a DA experienced less decisional conflict, were more likely to make a decision, and were more satisfied with their choice [28]. However, only a few DAs are currently available worldwide for individuals considering genetic testing for hereditary CRC [26]. To our knowledge, only 1 study [23] has examined the effectiveness of a tailored DA specifically designed to support individuals in making informed decisions about genetic testing for hereditary nonpolyposis CRC risk. The results indicated that the DA was effective in reducing uncertainty and helping individuals make an informed choice following genetic counseling. However, it had no significant impact on postdecisional regret or the actual uptake of genetic testing.

Individuals with different inherited cancer syndromes face varying risks of developing different types of cancer [2,3,29]. Moreover, for individuals carrying gene mutations associated with different inherited cancer syndromes, the strategies for reducing cancer risk also differ [2,3,29]. Therefore, DAs tailored to specific hereditary cancer syndromes may have different effects on individuals’ genetic counseling and genetic testing behaviors. To contribute further evidence to this field and to promote informed decision-making, increase the uptake of genetic testing among at-risk individuals, and ensure the rational use of genetic service resources, we developed a DA tool specifically for hereditary CRC genetic testing and compared its effect with treatment-as-usual in this randomized trial. The DA tool is suitable for patients with CRC, their relatives, and individuals considering genetic screening. In this study, we provided the DA tool to family members to promote the uptake of genetic screening among patients with CRC and delivered sufficient genetic information to support family members in making informed decisions. There were 2 main reasons for selecting family members, rather than patients, as research participants. First, most patients were unaware of their condition, and family members preferred to avoid discussing cancer-related information in their presence to prevent additional psychological burdens. Second, in line with practices in some other countries [30], physicians in China often communicate with family members about the patient’s medical condition and treatment options based on the principle of protective medicine [31]. This approach aims to mitigate the potential impact of psychological vulnerability and anxiety on treatment outcomes by selectively withholding certain details of the condition or treatment options from patients in specific clinical circumstances [32]. The inclusion of family members as research participants in this study aligns with the principle of protective medicine and relevant legal provisions, specifically Article 1219 of China’s Civil Code [33]. According to this article, medical professionals are required to provide clear explanations of medical conditions and treatment options to patients. However, when direct disclosure is deemed inappropriate—such as in cases where it may compromise the patient’s psychological well-being—medical professionals are permitted to communicate this information to the patient’s close relatives instead. This approach ensures that informed consent is obtained while also considering the patient’s best interests and adhering to ethical and legal standards [32]. To safeguard patient autonomy and uphold the right to informed consent, family members were instructed that they could disclose genetic screening information to patients at an appropriate time during the study period, based on the patient’s awareness of their condition and psychological status. Furthermore, delivering genetic information to family members typically does not cause psychocognitive burden or secondary trauma. Our previous research showed that family members reported they could accept information related to hereditary CRC and genetic testing without experiencing psychological burden [9]. A randomized trial by Rodriguez et al [34] also indicated that remote genetic education and testing services did not negatively affect cancer worry, anxiety, or depression scores among family members of patients with pancreatic cancer. In our study, spouses, biological siblings, and children were included. Given the important role of spouses within the family—and the fact that they share children with the patient—we believe that spouses, like biological siblings and children, play a significant role in the decision-making process regarding genetic testing for patients. Additionally, patients typically remained hospitalized for only 4-7 days, as the study unit followed a rapid rehabilitation surgical protocol. Therefore, the DA intervention was delivered via WeChat (Tencent Holdings Limited), a method that has been widely used and shown to be both feasible and effective [35,36]. As the participants in this study were family members of patients, we measured outcomes including informed decision-making, CRC screening behavior, healthy lifestyle scores (HLSs), anxiety and depression levels, quality of life, and satisfaction with the intervention. As the study focused on patients’ genetic testing, we also assessed their uptake of genetic counseling and testing. We hypothesized that the DA would promote informed decision-making—measured by knowledge, decisional conflict, self-efficacy, and preparation for decision-making—among family members regarding the genetic testing of patients with CRC. We also compared the 2 study arms in terms of patients’ uptake of genetic counseling and genetic testing, participants’ psychosocial outcomes, and satisfaction with the intervention. CRC screening, surveillance, and adopting a healthy lifestyle can reduce the incidence of CRC. Therefore, this study also examined whether the CRC prevention education component of the DA tool could promote participants’ CRC screening behaviors and the adoption of a healthy lifestyle.

## Methods

### Study Design and Participants

An assessor-blinded, 2-arm randomized controlled trial with repeated measures and parallel groups was conducted in China, comparing participants who received the 6-week DA intervention with those in a waiting list control group. This study was reported in accordance with the CONSORT (Consolidated Standards of Reporting Trials) statement for reporting parallel-group trials [37] and the TIDieR (Template for Intervention Description and Replication) guideline.

From July to August 2021, 1 family member of each patient with CRC was recruited from the gastric and colorectal surgery unit of a top-tier general hospital in Changchun, Jilin Province, China, which provided high-quality medical services and facilities for our research. Recruitment was conducted by the researchers and nurses in the unit. Potential participants were given written and verbal information about the study, along with the informed consent form and baseline survey. On the day of the patient’s discharge, researchers or nurses followed up with potential participants who had not yet completed the baseline survey. After completing the baseline survey, the principal researcher (HL) added the WeChat accounts of consenting participants. Eligible participants met the following criteria: aged 18 years or older; a family member of a patient diagnosed with colon or rectal cancer; normal auditory and visual ability, with the capacity to read and communicate with the researcher in Chinese; acted as a decision maker for the patient’s treatment and testing; neither the patient nor the family member had previously received genetic counseling or genetic testing; owned and were able to use a smartphone or tablet with internet access and the WeChat mobile app; and voluntarily agreed to participate in the study. Participants with severe physical or mental health conditions or a history of psychotic illness were excluded.

### Ethical Considerations

The trial was approved by the Human Research Ethics Committee of the School of Nursing, Jilin University (approval number 2021062501), and registered in the Chinese Clinical Trial Registry (ChiCTR2100048051) before study commencement. There were 2 deviations from the registered protocol: (1) participants were recruited from 1 hospital instead of 2, as originally planned, due to COVID-19 restrictions; and (2) we did not provide educational materials to participants in the control group, and instead compared the effectiveness of the DA intervention with usual care (as opposed to DA versus education, as stated in the protocol). This adjustment was made based on expert recommendations emphasizing the importance of comparing DA with usual care, and due to limited workforce and funding, which made it infeasible to conduct a 3-arm study involving DA, education, and usual care at that time. The DA tool was provided to participants in the control group after the study concluded. All participants gave written informed consent before enrollment. They were informed that participation was entirely voluntary, that all collected data would remain confidential, and that they could decline or withdraw from the study at any time without affecting their medical care or relationship with health care providers. Each participant was assigned a numerical identifier for data entry, storage, and analysis, and all personal information was anonymized. Baseline data (T0) were stored by the institutional review board. Data from the online surveys (T1 and T2) were stored on the Questionnaire Star server (operated by Changsha Ranxing Information Technology Co., Ltd.) and secured with a password. To protect patient privacy and confidentiality, only research staff approved by the institutional review board had access to the data. No compensation was provided to participants for their involvement in the study.

### Randomization and Procedure

After completing the baseline survey (T0), participants were randomly assigned in a 1:1 ratio to either the DA intervention group or the treatment-as-usual group using a computer-generated randomization sequence. Group allocation codes were generated based on this sequence, with “1” indicating the DA intervention group and “2” representing the control group. These codes were placed into sequentially numbered, opaque, and sealed envelopes. The researcher (JS) who generated the random number sequence and prepared the envelopes was not involved in participant recruitment, group allocation, intervention delivery, or data collection. Another researcher (WL), who also did not participate in intervention delivery or data collection, assigned participants to groups based on their enrollment order and the corresponding group codes in the numbered, opaque, sealed envelopes. Approximately 6 weeks (T1) and 3 months (T2) after randomization, participants completed the postintervention and follow-up surveys. The DA intervention and outcome assessments at T1 and T2 were conducted via WeChat. We used Questionnaire Star (Changsha Ranxing Information Technology Co., Ltd.), a widely adopted web-based survey platform in China, to develop the follow-up questionnaires. At both time points, participants received a link to the questionnaire via their individual WeChat accounts. To prevent duplicate responses, each account was permitted to access the questionnaire only once. The survey consisted of 94 items, organized over 6 pages with approximately 16 items per page, and required about 20 minutes to complete. The Questionnaire Star platform performed automatic completeness checks before submission, and participants could review or modify their responses using the Back button. Before each assessment, researchers who had received standardized training contacted participants via WeChat voice calls to explain the purpose and content of the questionnaire, as well as instructions for completing the online survey. The outcome assessors were blinded to group allocation. However, due to the nature of the intervention and the absence of an active control condition, it was not feasible to blind the principal researcher delivering the DA intervention or the participants. Both the researcher and participants were therefore aware of group assignments throughout the study.

### DA Tool Development and Intervention

The DA tool was developed by nursing researchers in collaboration with colorectal oncologists, gastric and colorectal surgeons, and nurses from oncology and gastrointestinal surgical units. Its development was informed by the needs of patients with CRC and their family members, existing evidence from the literature and clinical guidelines, the International Patient Decision Aid Standards, and the Ottawa Decision Support Framework [38]. Additionally, feedback from CRC clinical experts gathered through a Delphi expert consultation process was incorporated into the tool’s design (see Multimedia Appendix 1). The DA provided balanced information on 2 options: undergoing genetic testing or not undergoing testing. The DA material comprised 40 pages. Based on feedback from CRC clinical experts, the content was organized into 6 topics aligned with tertiary prevention strategies for CRC and the Ottawa Decision Support Framework (see Multimedia Appendix 2). Topics 1-5 presented evidence-based information on hereditary CRC-related cancer risks; the potential benefits, risks, and limitations of genetic testing; possible test outcomes; the potential impact of testing on individuals and their families; and cancer prevention strategies tailored to individuals at varying levels of CRC risk. Topic 6 concluded the DA with 9 patient stories, illustrating the perspectives and decision-making processes of patients and family members regarding CRC genetic evaluation. Additionally, a blank personal worksheet (a value clarification exercise) was included to help individuals list and rate the importance of the pros and cons of genetic testing in their own context. To enhance the accessibility and visual appeal of the DA content, the material was simplified and adapted into 4 animated videos with a total runtime of 10 minutes and 37 seconds. The relationship between the PDF materials and the video content is presented in Multimedia Appendix 3. A more detailed description of the DA tool can be found in Li [9].

To reduce participant burden and enhance adherence, the principal researcher (HL) delivered 1 DA topic per week over 6 weeks in PDF (6 topics, 6 PDF files). Each PDF file was accompanied by a corresponding animated video, both sent to participants via WeChat (see Multimedia Appendix 3). Participants were encouraged to view the materials at their own pace using their personal mobile devices. To support engagement, the research team sent weekly notifications to introduce each new topic and reminders to review any unfinished content via WeChat throughout the 6-week intervention and the subsequent 6-week follow-up period. The researcher maintained regular contact with participants throughout the intervention and follow-up periods. Adherence was assessed biweekly. Participants in the intervention group received follow-up phone calls or WeChat messages to monitor their progress with the online learning materials, answer any questions, and address any obstacles they encountered. The principal researcher (HL), a registered nurse with a PhD in nursing, had received specialized training in CRC genetic evaluation. Before the study, the researcher further strengthened their competency through on-site discussions with colorectal oncologists and nurse managers, as well as by acquiring up-to-date knowledge and skills from online resources, academic conferences, and peer-reviewed journals.

### Treatment as Usual

Participants in both groups received the standard care routinely provided by the study site. In China, cancer-related genetic screening remains in its early stages. Clinicians typically inquire about family history following patient admission. However, the advice and support offered to patients and their families regarding genetic evaluation largely depend on the clinicians’ perspectives—such as their attitudes toward genetic testing for hereditary CRC—and on patient and family characteristics, including health literacy and financial status.

### Measures

#### Sociodemographic and Clinical Variables

Participants’ demographic characteristics, including age, gender, education level, and relationship with the patient, were assessed at baseline (T0). In addition, information regarding the patient’s disease diagnosis, family history of cancer, type of medical insurance, and level of cancer-related financial distress was collected.

#### Knowledge

Genetic knowledge was assessed at T0, T1, and T2 using a self-designed 16-item questionnaire. The development of the questionnaire was guided by the framework of the knowledge questionnaire from the Ottawa Patient Decision Aids website [39], informed decision-making measurement tools [40], and relevant literature [41-48]. The questionnaire was revised and refined through 2 rounds of expert consultation and a pilot survey. It evaluates respondents’ understanding of hereditary CRC and genetic testing, including its alternatives, rationale, key benefits, risks, and potential side effects. All items were answered using a true/false/unclear format, with the “unclear” option included to minimize guessing. The total knowledge score was calculated by summing the number of correct responses.

#### Decisional Conflicts, Decision Self-Efficacy, and Preparation for Decision-Making

Decisional conflict and self-efficacy regarding the genetic testing decision were assessed at T0, T1, and T2 using the Decisional Conflict Scale (DCS) [49] and the Decision Self-Efficacy Scale [50], respectively. Preparation for decision-making was measured at T1 and T2 using the Preparation for Decision Making Scale [51]. The DCS includes 4 subscales that assess modifiable factors contributing to decisional conflict: feeling informed, clarity of personal values, perceived support in decision-making, and confidence in the decision made. The Decision Self-Efficacy Scale measures an individual’s confidence or belief in their ability to make health-related decisions. The Preparation for Decision Making Scale evaluates the respondent’s perception of how helpful a DA intervention is in preparing them to communicate effectively with a health care provider during a consultation focused on decision-making. A score below 60 indicates inadequate preparation for decision-making. The Chinese versions of these 3 scales are available for free use on the Ottawa Patient Decision Aids website [52] and demonstrated satisfactory internal consistency in this study, with Cronbach α coefficients of 0.973, 0.952, and 0.999, respectively.

#### Genetic Counseling and Testing

Patients’ genetic counseling and testing uptake were assessed at T1 and T2 using 2 self-developed questions: (1) Has the patient or someone else (on behalf of the patient) consulted a health care professional regarding hereditary CRC and genetic testing? and (2) Has the patient undergone genetic testing?

#### CRC Screening and Healthy Lifestyle

CRC screening behavior was assessed at T0, T1, and T2 using a self-developed question: “What type of CRC screening have you undergone?”

HLSs [53] were assessed at T0, T1, and T2. The HLSs were constructed based on the 8 variables listed in Textbox 1.

**Variables used for the construction of healthy lifestyle scores.**

- BMI (kg/m^2^): 18.5-23.9 kg/m^2^ (based on Chinese criteria)
- Waist circumference: <80 cm for women and <85 cm for men (Chinese standard used instead of waist-to-hip ratio for easier measurement)
- Physical activity: 150-300 minutes per week of moderate-intensity activity or 75-150 minutes per week of vigorous-intensity activity, or an equivalent combination
- Sedentary behavior: leisure-time sedentary activities (eg, watching television, using a computer) <3 hours per day
- Processed and red meat intake: <4 times per week
- Vegetable and fruit intake: >5 servings per day (1 serving=80 g)
- Alcohol consumption: never or seldom (only on special occasions or 1-3 times per month)
- Tobacco use: never smokers

Each variable was assigned a score of 0 or 1, with 1 indicating a healthy behavior. The total HLSs ranged from 0 to 8. For analysis, participants were categorized into 3 groups based on their total HLSs: unhealthy (scores 0-1), intermediate (scores 2-3), and healthy (scores ≥4).

#### Anxiety and Depression and Quality of Life

The validated Chinese versions of the Hospital Anxiety and Depression Scale (HADS) and the 12-Item Short-Form Health Survey (SF-12) were used to assess anxiety, depression, and quality of life at T0, T1, and T2. The HADS includes 2 subscales: a 7-item anxiety subscale and a 7-item depression subscale. The SF-12 assesses 2 components of health-related quality of life: the physical component summary and the mental component summary. Both instruments have demonstrated high internal consistency and strong structural validity in previous studies [54,55].

#### Satisfaction With the Intervention

Satisfaction with the intervention was assessed at T2 using 4 self-designed items, covering the following aspects: overall satisfaction, adequacy of the intervention content, intelligibility of the content, and satisfaction with the WeChat-based information delivery method.

### Statistical Analyses

Data analyses were conducted using IBM SPSS Statistics for Windows, version 25.0. An intention-to-treat analysis was performed using the last observation carried forward method to impute missing data based on baseline responses, thereby minimizing selection bias. Continuous variables are presented as means with SDs or medians with IQRs, while categorical variables are presented as frequencies and percentages. Differences between the 2 groups were analyzed using the independent samples 2-tailed *t* test, Mann-Whitney *U* test, chi-square test, or Fisher exact test, as appropriate. The McNemar test was used for intragroup comparisons. A generalized estimating equation model was used to compare repeated measures outcomes across the time points between the 2 groups. Statistical significance was set at *P*<.05 (2-tailed). Effect sizes were calculated using Cohen *d* for continuous variables and Cramér V or phi for categorical variables. As there were no missing data among participants who completed the T1 and T2 surveys, the missing data rate was equal to the dropout rate. Sensitivity analyses were conducted using a per-protocol approach, which excluded participants with any missing data components, to assess the potential impact of attrition on the study outcomes. Based on the effect size (Cohen *d*=0.81) for decisional conflict scores reported by Wakefield et al [23], a total sample size of 64 participants (32 per group) was estimated to achieve a statistical power of 0.90 at a 2-sided significance level of .05. Considering an anticipated attrition rate of 20%, the target sample size was increased to 80 participants (40 per group) for this study.

## Results

### Overview

**Figure 1:**
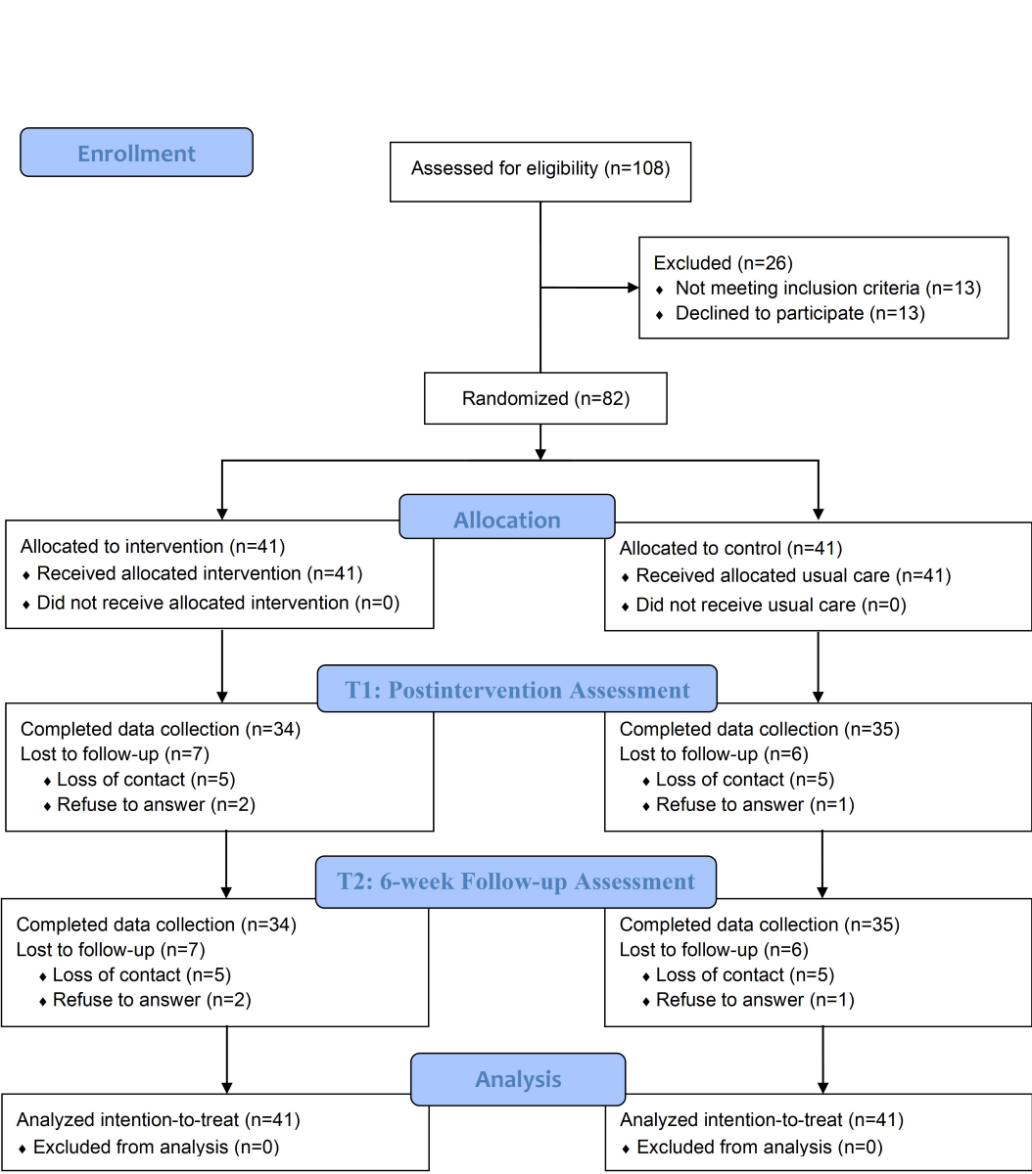
Study flowchart.

A total of 108 participants were assessed for eligibility, of whom 26 were excluded. The remaining 82 participants were then randomized to either the DA intervention group or the treatment-as-usual group. Of these 82 participants, 69 completed both the T1 and T2 assessments, while 13 dropped out due to unwillingness to continue participation or loss of contact (Figure 1 and Multimedia Appendices 4-6). Baseline demographic characteristics of participants, along with patients’ clinical characteristics and family history, are presented in Multimedia Appendix 7. No significant differences were observed between groups at baseline (Multimedia Appendices 7 and 8), except for the patient’s disease diagnosis (colon vs rectal cancer), which showed a statistically significant difference (*P*=.047). We believe that the difference in disease diagnosis is unlikely to have affected the study outcomes. No statistically significant differences were found in baseline characteristics between participants who completed the study and those who withdrew ( Multimedia Appendix 9). Sensitivity analyses using a per-protocol approach yielded results consistent with the primary analysis, suggesting that missing data had minimal impact on the findings. By the end of the study, 4 out of 41 (10%) participants in the DA group reported not engaging with the PDF materials or animated videos (Multimedia Appendix 10).

### Effects of the DA Tool on Study Outcomes

#### Primary Outcomes: Knowledge, Decisional Conflicts, Decision Self-Efficacy, and Preparation for Decision-Making

Compared with the baseline, both groups demonstrated increased knowledge and decision self-efficacy scores, along with decreased decisional conflict scores (see Multimedia Appendix 11). The generalized estimating equation model revealed a statistically significant group × time interaction effect on knowledge, decisional conflict, and decision self-efficacy (*P*<.001 for all; Table 1). At T1 and T2, 16 (39%) and 20 (49%) of the 41 participants in the intervention group, respectively, reported good preparation for decision-making (Multimedia Appendix 12).

**Table 1:** Generalized estimating equation models of the comparison of study outcomes between the intervention and control groups.

Variables	Group effect		Time effect			Group × time effect	
	Wald chi-square (*df*)	*P* value	Wald chi-square (*df*)	*P* value	Wald chi-square (*df*)		*P* value
Knowledge	7.158 (1)	.007	44.719 (2)	<.001	35.772 (2)		<.001
Decisional conflicts	12.296 (1)	<.001	53.89 (2)	<.001	32.283 (2)		<.001
Decision self-efficacy	7.066 (1)	.008	55.81 (2)	<.001	42.692 (2)		<.001
Physical component summary	0.559 (1)	.46	1.48 (2)	.48	1.778 (2)		.41
Mental component summary	0.179 (1)	.67	0.687 (2)	.71	0.726 (2)		.70
Anxiety	0.036 (1)	.85	0.987 (1)	.32	0.941 (1)		.33
Depression	3.008 (1)	.08	0.987 (1)	.32	0.94 (1)		.33
Colorectal cancer screening in 5 years	4.566 (1)	.03	16.624 (2)	<.001	1.093 (2)		.58
Tobacco smoking	0.003 (1)	.95	2.007 (1)	.16	0.147 (1)		.70
Alcohol consumption	0.502 (1)	.48	0.978 (2)	.61	2.921 (2)		.23
BMI (kg/m^2^)	1.194 (1)	.27	1.071 (2)	.59	1.053 (2)		.59
Waist circumference	0.211 (1)	.65	2.047 (1)	.15	0.001 (1)		.97
Physical activity	0.053 (1)	.82	2.048 (1)	.15	0 (1)		.99
Sedentary time (hours/day)	0.255 (1)	.61	7.824 (1)	.005	1.271 (1)		.26
Processed and red meat intake	0.284 (1)	.59	1.024 (1)	.31	1.024 (1)		.31
Vegetable and fruit intake	0.68 (1)	.41	6.419 (1)	.01	0.135 (1)		.71
Healthy lifestyle scores	0.526 (1)	.47	5.255 (2)	.07	6.463 (2)		.04

#### Secondary Outcomes: Genetic Counseling and Testing

At T2, 2 (5%) participants in each group (n=41 per group) reported consulting a genetic counselor on behalf of the patient. Additionally, 2 patients (5%) in the DA group and 1 patient (2%) in the treatment-as-usual group underwent hereditary CRC genetic testing. No statistically significant differences were observed between the groups (*χ*^2^_1=1_=0, *P*>.99; see Multimedia Appendix 12).

#### Secondary Outcomes: CRC Screening and Healthy Lifestyle

A statistically significant group × time interaction effect was observed for HLSs (*P*=.04; Table 1). Specifically, a significant interaction effect was found at T2 (β=1.037, *P*=.03), but not at T1. Among the individual components of the HLSs, a statistically significant group × time interaction was noted for processed and red meat intake at T1 (β=–1.494, *P*<.001), while no significant difference was found at T2 (β=–.107, *P*=.31). A statistically significant time effect was observed for CRC screening (T1: β=.726, *P*=.003; T2: β=1.123, *P*<.001), vegetable and fruit intake (T1 and T2: β=.473, *P*=.01), and sedentary time (T1 and T2: β=–.122, *P*=.005). However, no significant group effect or group × time interaction effect was detected for these outcomes. For detailed results, see Multimedia Appendix 13.

#### Secondary Outcomes: Anxiety, Depression, and Quality of Life

No statistically significant group × time interaction effects were observed for anxiety (*P*=.33), depression (*P*=.33), or quality of life (physical component summary: *P*=.41; mental component summary: *P*=.70; Table 1). Similarly, no significant group effects or time effects were detected for these outcomes (group effects of anxiety: *P*=.85; time effects of anxiety: *P*=.32; group effects of depression: *P*=.08; time effects of depression: *P*=.32; group effects of physical component summary: *P*=.46; time effects of physical component summary: *P*=.48; group effects of mental component summary: *P*=.67; and time effects of mental component summary: *P*=.71, Table 1).

#### Satisfaction With the Intervention

Except for participants who were lost to follow-up and those who did not engage with the DA materials, most participants in the DA group reported that the intervention content was understandable, comprehensive, and sufficient. They also expressed satisfaction with the WeChat-based delivery method (see Multimedia Appendix 14).

## Discussion

### Principal Findings

The results demonstrated that the DA intervention had positive effects on improving participants’ knowledge, reducing decisional conflict, enhancing decision self-efficacy, and increasing preparation for decision-making. These findings are consistent with previous research, which suggested that a computer-based DA was more effective than standard genetic counseling in increasing knowledge about breast cancer and reducing the intention to undergo genetic testing among women at low risk of carrying a *BRCA1/2* mutation [56]. Another study indicated that a tailored DA could reduce uncertainty and support individuals in making informed decisions about genetic testing for hereditary nonpolyposis CRC following genetic counseling [23]. Taken together, the evidence suggests that DA programs have the potential to serve as effective educational interventions for individuals at low risk, and can also supplement clinical genetic counseling—particularly in busy oncology settings where formal counseling may be limited or unavailable for those at high risk. It is important to note, however, that our study specifically evaluated the effectiveness of the DA tool in promoting informed decision-making among family members regarding genetic testing for patients with CRC. Consistent with previous studies demonstrating the benefits of DA interventions for both affected individuals and high-risk, unaffected relatives [23], we anticipate that our DA tool will also facilitate informed decision-making regarding genetic testing among high-risk family members of patients diagnosed with hereditary CRC. This hypothesis will be further explored and validated in our future research.

At T2, 3 patients underwent hereditary CRC genetic testing—2 from the DA group and 1 from the treatment-as-usual group. Notably, only 1 patient from the DA group received genetic testing with the support of the DA tool. According to family members, the other 2 patients pursued genetic testing based on their clinicians’ recommendations. Beyond clinical guidance, another potential reason for the lack of significant difference in genetic evaluation uptake between the 2 groups may be the relatively short follow-up period in this study. Most patients were in the postoperative rehabilitation or chemotherapy phase, during which family members tended to prioritize immediate treatment concerns over genetic evaluation. Despite the low uptake of genetic testing, participants in the DA group demonstrated significantly higher levels of knowledge, decision self-efficacy, and preparation for decision-making, along with lower decisional conflict. These findings may reflect more informed decision-making and a more accurate perception of cancer risk, particularly among participants assessed as having a low genetic risk. A previous study also found that DAs did not influence participants’ actual decision-making or genetic testing uptake. However, participants identified as having low genetic risk demonstrated more accurate perceptions of their risk and lower intentions to pursue genetic testing following the intervention [56]. This outcome supports the rational allocation of genetic services by helping to avoid unnecessary testing among low-risk individuals.

A statistically significant time effect was observed for CRC screening, vegetable and fruit intake, and sedentary time, while no significant group or group × time interaction effects were found. These findings suggest that these variables improved over time regardless of group assignment, indicating that the DA intervention may not provide significant advantages over usual care in these areas. A previous qualitative study reported that clinicians often recommend colonoscopy for patients’ family members [9], which may have contributed to the observed improvements in CRC screening across both groups. The reduction in sedentary time may be attributed to participants being occupied with caregiving responsibilities for patients during the postoperative period. For the other components of the HLSs, no significant time or group effects were observed. This may be explained by the fact that most participants were nonsmokers, consumed alcohol fewer than 3 times per week, and already met recommended physical activity levels at baseline. Furthermore, lifestyle modification is a gradual and often challenging process. Although previous research has shown participants’ willingness to adopt healthier behaviors [57], the 6-week follow-up period in this study may have been too short to observe significant changes. Without sustained support and longer-term follow-up, short-term interventions may be insufficient to produce and maintain lasting lifestyle improvements. Further research is needed to evaluate the long-term impact of CRC education on health behavior change.

The potential negative psychological impact of genetic testing for hereditary cancers has long been a concern among geneticists and researchers. However, in this study, no significant differences were observed between groups in any of the psychosocial outcomes. This finding aligns with our previous qualitative research, which indicated that family members felt capable of accepting information related to hereditary CRC and genetic testing without experiencing psychological distress [9]. Similarly, Esteban et al [58] reported that patients were able to psychologically cope with cancer panel testing. Only 10%-20% of individuals who undergo genetic counseling experience serious psychological issues after learning they carry a familial mutation gene. Some may report heightened cancer-related concerns in the weeks following the receipt of test results. However, such concerns are often linked to preexisting anxiety about cancer, elevated perceived risk, and a positive genetic test outcome [58]. These findings underscore the importance of providing additional support during genetic counseling, particularly for individuals who exhibit high levels of cancer worry, perceive themselves to be at elevated risk, or demonstrate limited social coping skills [59].

Most participants in the DA group who engaged with the content reported high levels of satisfaction. However, 4 out of 41 (10%) participants did not read the DA materials at T2, citing temporary responsibilities in caring for patients. Additionally, 7 (17%) participants reported only briefly reviewing the content or focusing on sections they found relevant. A few participants noted that the DA content was too lengthy or difficult to understand. These findings suggest opportunities for optimizing the DA tool, including simplifying the content, identifying more suitable intervention timing, and extending the follow-up period to better accommodate participants’ needs.

### Limitations

This study had several limitations. First, all participants were recruited from a single hospital, which may limit the generalizability of the findings to other regions or countries. Second, although family members were included as research participants based on the principles of preventive medicine and relevant legal provisions, and were encouraged to share the study content with patients at appropriate times, by the end of the follow-up period, approximately half had not informed the patients about the intervention or discussed genetic screening decisions with them. The effectiveness of the DA for patients themselves, their willingness to participate in genetic screening decisions, and particularly their perspectives on family-based decision-making regarding genetic testing warrant further investigation. Third, while the current DA presents balanced information on 2 options—undergoing testing and not undergoing testing—future versions could incorporate a third option: delayed testing. This addition would help identify and support participants who may be temporarily unable to consider CRC genetic evaluation due to caregiving responsibilities. Fourth, given the small sample size and the use of a spontaneous and untreated usual care group, further research is necessary to validate these findings and to assess the DA’s effectiveness compared with an active control group. Moreover, all outcomes in this study were assessed through self-reported questionnaires, without the use of objective measurements. This reliance on subjective assessments may introduce bias, particularly in reporting actual genetic counseling and genetic testing uptake. Additionally, the follow-up duration was limited to 6 weeks due to constraints in workforce and time, which restricts the ability to evaluate the long-term effects of the intervention—especially regarding genetic evaluation uptake and CRC prevention behaviors. Finally, participant characteristics such as age, educational level, and perceived cancer risk may influence the intervention’s effectiveness. Further research is necessary to explore how these factors may affect outcomes and to guide the future implementation of the intervention in broader clinical settings.

### Implications for Practice and Research

The findings of this study suggest that the DA intervention has the potential to enhance informed decision-making among family members regarding genetic screening for patients with CRC and to support appropriate uptake of genetic testing. Delivered via WeChat—the most widely used social media platform in China—our intervention is seamlessly integrated into participants’ daily routines. Web-based interventions such as this reduce perceived barriers and participation burdens, making them more accessible and user-friendly. Notably, this pragmatic, clinic-integrated online program builds on existing clinical resources and evidence related to CRC genetic screening. These findings can inform the future refinement of DA interventions and support the broader implementation of genetic screening programs in clinical practice.

To support the successful implementation of such interventions among patients with CRC and their family members, several practical and clinical considerations are essential. First, the active involvement of health care professionals—including nurses, physicians, geneticists, and oncologists—is critical. Second, these professionals should receive training to serve as primary facilitators of genetic screening and informed decision-making interventions. Among them, nurses play a particularly vital role by providing ongoing information, emotional support, and follow-up for individuals at increased genetic risk. This study underscores the competence and pivotal role of nurses in cancer genetic evaluation—a contribution that is often underrecognized in clinical practice. Our review found that only 2 out of 8 studies included nurses as intervention providers to conduct familial cancer risk assessments and deliver education about Lynch syndrome [26]. Moving forward, it is essential that more nurses receive specialized genetics training to effectively participate in genetic evaluation efforts. Their involvement should extend to collaboration with other stakeholders in the development, implementation, and evaluation of cancer genetic screening programs. This will help improve genetic referrals for individuals at risk. Third, personalized risk assessment plays a critical role in motivating high-risk patients and their families to actively engage in genetic screening. Integrating individualized risk assessments for every patient with CRC into future DA interventions may enhance user engagement and the overall impact of these programs. Fourth, education on CRC prevention has the potential to encourage healthier behaviors among participants. Health care providers can play a key role by assessing individuals’ health behaviors during the genetic screening process and offering targeted interventions to those with unhealthy lifestyles or inadequate screening practices. This integrated approach not only supports risk management for individuals with a high genetic predisposition but also contributes to broader efforts in CRC prevention within the general population.

The findings of this study offer important guidance for optimizing the DA tool. Specifically, there is a need to develop a more streamlined, intelligent, and user-friendly version that can automatically track participants’ progress, including the number of topics completed and frequency of visits. Additionally, the tool should include features to prevent fast-forwarding or skipping content, ensuring full engagement with the material. The results also underscore the importance of refining the intervention implementation protocol to enhance its effectiveness and usability. Given the various limitations of this study—including its single-center design, short follow-up period, lack of an active control group, and reliance on self-reported data—further randomized controlled trials are necessary to validate the effectiveness of the DA tool. Future research should address these limitations by incorporating multicenter designs, extended follow-up periods, objective outcome measurements, and active comparators to generate more robust evidence for the implementation of DA tools in genetic screening decision-making.

### Conclusions

Our findings suggest that the DA intervention may be a safe and effective approach to promote informed decision-making among family members regarding the genetic testing of patients with CRC. The developed DA tool has the potential to serve as a valuable adjunct to existing cancer genetic evaluation practices. However, there is a need to develop and test more simplified, user-friendly, and widely accessible DA tools to enhance their usability and impact.

## Appendix

Multimedia Appendix 1 Evidence for the development of the decision aid tool.

Multimedia Appendix 2 Decision aid framework.

Multimedia Appendix 3 Decision aid intervention procedures.

Multimedia Appendix 4 CONSORT (Consolidated Standards of Reporting Trials) checklist.

Multimedia Appendix 5 TIDieR (Template for Intervention Description and Replication) checklist.

Multimedia Appendix 6 CHERRIES (The Checklist for Reporting Results of Internet E-Surveys) checklist.

Multimedia Appendix 7 Baseline sociodemographic/clinical characteristics and differences between groups.

Multimedia Appendix 8 Baseline study outcomes and differences between groups.

Multimedia Appendix 9 Differences in baseline characteristics between participants who completed the study and those who withdrew.

Multimedia Appendix 10 Participants’ adherence to the intervention.

Multimedia Appendix 11 Outcomes of the 2 groups at T1 and T2.

Multimedia Appendix 12 Comparison of preparation for decision-making, genetic counseling, and genetic testing between T1 and T2.

Multimedia Appendix 13 Generalized estimating equation models of the comparison of study outcomes between the intervention and control groups.

Multimedia Appendix 14 Satisfaction with the decision aid intervention.

## Abbreviations

CONSORT: Consolidated Standards of Reporting Trials
CRC: colorectal cancer
DA: decision aid
DCS: Decisional Conflict Scale
HADS: Hospital Anxiety and Depression Scale
HLS: healthy lifestyle score
NCCN: National Comprehensive Cancer Network
SF-12: 12-Item Short-Form Health Survey
TIDieR: Template for Intervention Description and Replication

## Data Availability

The raw data supporting the conclusions of this article will be made available by the authors upon reasonable request.
