# Supplementary material for: A WeChat-Based Decision Aid Intervention to Promote Informed Decision-Making for Family Members Regarding the Genetic Testing of Patients With Colorectal Cancer: Randomized Controlled Trial
Source: J Med Internet Res. 2025 Apr 21;27:e60681. doi: 10.2196/60681 (PMC12053134; doi:10.2196/60681)
Supplement: Multimedia Appendix 1 [file jmir_v27i1e60681_app1.docx]

| **Appendix 1 Evidence for the development of the decision aid tool.** | |
| --- | --- |
| **Categories** | **Title** |
| Ottawa Decision Support Framework and International Patient Decision Aid Standards. | |
| Decisional needs from patients with colorectal cancer and their relatives based on our previous studies. | |
| Opinions of colorectal cancer clinical experts based on the Delphi Expert Consultation. | |
| Research literature | 1. Randomized trial of a decision aid for individuals considering genetic testing for hereditary nonpolyposis colorectal cancer risk. |
| Guidelines | 1. NCCN Guidelines Version 1.2020：Genetic/Familial High-Risk Assessment: Colorectal. 2. NCCN Guidelines Version 2.2020: Colorectal Cancer Screening. 3. ACG Clinical Guidelines: Colorectal Cancer Screening 2021. 4. China Guideline for the Screening, Early Detection and Early Treatment of Colorectal Cancer (2020, Beijing). 5. American Cancer Society guideline for diet and physical activity for cancer prevention. 6. The Dietary Guidelines for Chinese Residents (2016). 7. World Health Organization 2020 guidelines on physical activity and sedentary behaviour. |
| Expert consensus | 1. The Chinese expert consensus on clinical diagnosis, treatment and pedigree management of hereditary colorectal cancer. 2. Consensus of Chinese experts on clinical detection of molecular markers of colorectal cancer. 3. Chinese consensus of early colorectal cancer screening (2019, Shanghai). 4. Consensus on the detection of microsatellite instability in colorectal cancer and other related solid tumors in China. 5. Chinese Consensus on Prevention of Colorectal cancer (2016, Shanghai). 6. The World Cancer Research Fund/American Institute for Cancer Research Third Expert Report on Diet, Nutrition, Physical Activity, and Cancer: Impact and Future Directions. |
| Decision Aids published on website https://decisionaid.ohri.ca/index.html | 1. Understanding genetic tests for Lynch syndrome: Information and decision aid. Centre for Genetics Education, NSW Health. |

*Note.* NCCN: National Comprehensive Cancer Network; ACG: American College of Gastroenterology.
