# Supplementary material for: A WeChat-Based Decision Aid Intervention to Promote Informed Decision-Making for Family Members Regarding the Genetic Testing of Patients With Colorectal Cancer: Randomized Controlled Trial
Source: J Med Internet Res. 2025 Apr 21;27:e60681. doi: 10.2196/60681 (PMC12053134; doi:10.2196/60681)
Supplement: Multimedia Appendix 2 [file jmir_v27i1e60681_app2.docx]

**Appendix 2 Framework of Decision Aids.**


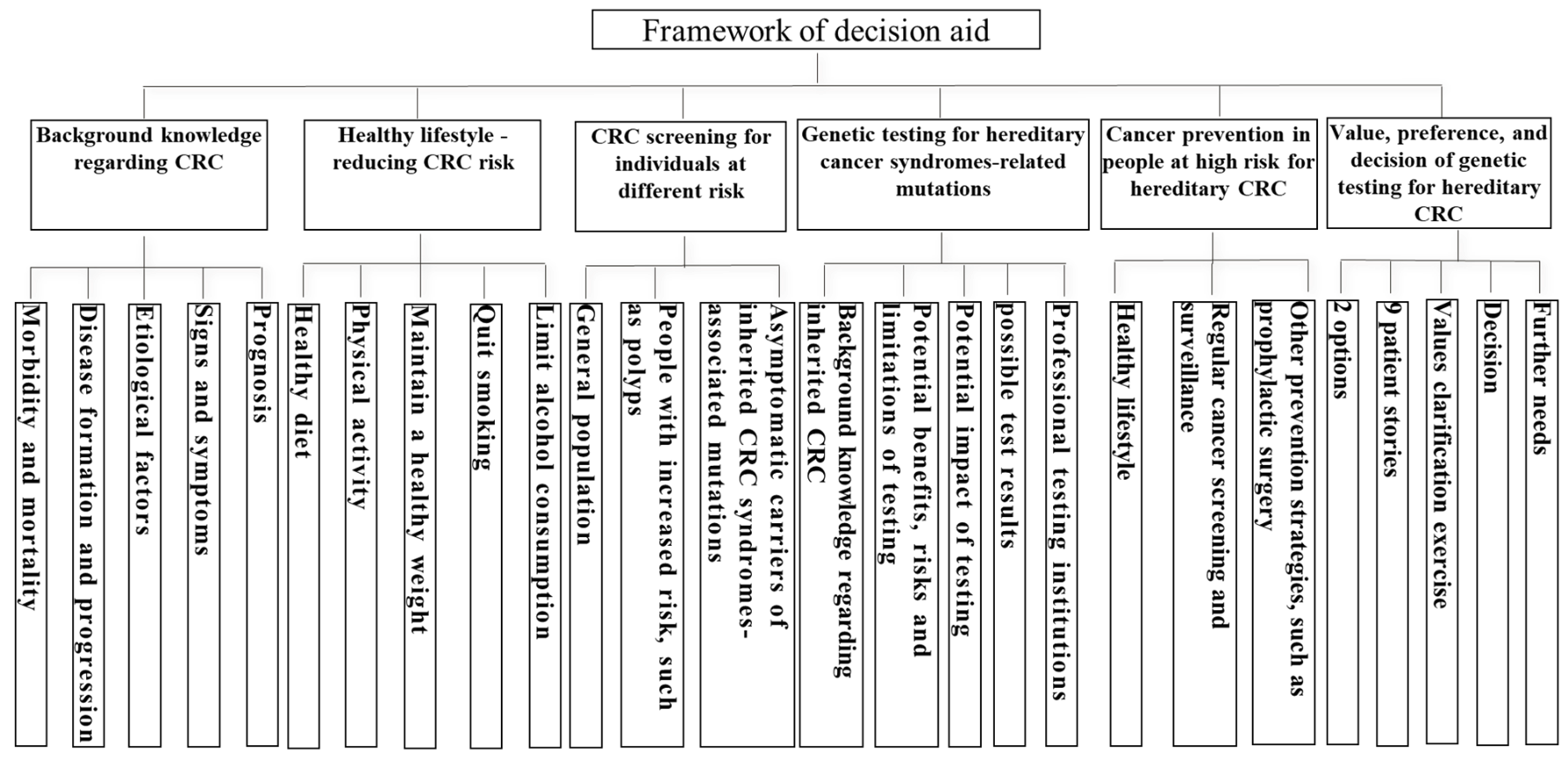


Note. We aim to provide knowledge and information support for family members to help them understand the risks and benefits of genetic screening by Topics 1-5. Topic 6 provides 9 stories about patients' or family members’ view and decision process about CRC genetic evaluation and a blank personal worksheet (value clarification exercise) to help participants clarify their values and preferences regarding the genetic screening of patients and promote participants' informed decision-making.
