# Supplementary material for: A WeChat-Based Decision Aid Intervention to Promote Informed Decision-Making for Family Members Regarding the Genetic Testing of Patients With Colorectal Cancer: Randomized Controlled Trial
Source: J Med Internet Res. 2025 Apr 21;27:e60681. doi: 10.2196/60681 (PMC12053134; doi:10.2196/60681)

**Appendix 3 Decision aid intervention procedures (PDF files 1-4 were made into videos 1-4, respectively. Part of PDF file 5 were duplicated with PDF file 2 and 3. The remaining contents of PDF file 5 is about other cancer prevention strategies, such as prophylactic surgery etc., which were added into the video 4.).**


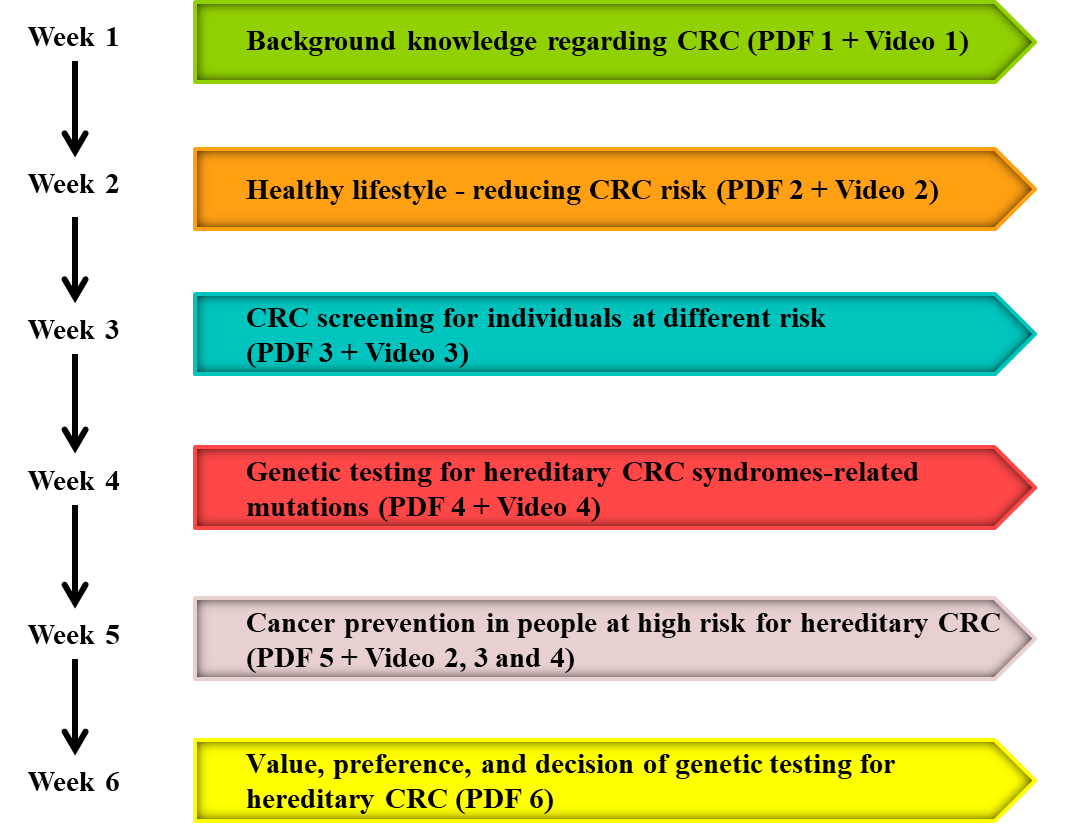

Supplement: Multimedia Appendix 3 [file jmir_v27i1e60681_app3.docx]
