# Supplementary material for: A WeChat-Based Decision Aid Intervention to Promote Informed Decision-Making for Family Members Regarding the Genetic Testing of Patients With Colorectal Cancer: Randomized Controlled Trial
Source: J Med Internet Res. 2025 Apr 21;27:e60681. doi: 10.2196/60681 (PMC12053134; doi:10.2196/60681)
Supplement: Multimedia Appendix 7 [file jmir_v27i1e60681_app7.docx]

**Appendix 11 Baseline sociodemographic/clinical characteristics and differences between groups.**

| **Variable** | **All (n=82)** | **Intervention (n=41)** | **Control (n=41)** | ***t/******χ^2^*** | ***P*** |
| --- | --- | --- | --- | --- | --- |
| **Age** | 42.39±10.91 | 42.05±10.61 | 42.73±11.32 | ﹣0.028^a^ | .78 |
| **Gender** |  |  |  |  |  |
| Male | 30（37%） | 14（34%） | 16（39%） | 0.21^b^ | .65 |
| Female | 52（63%） | 27（66%） | 25（61%） |  |  |
| **Education level** |  |  |  |  |  |
| High school and below | 35（43%） | 15（37%） | 20（49%） | 1.256 ^b^ | .53 |
| Secondary technical school/College degree | 25（30%） | 14（34%） | 11（27%） |  |  |
| Bachelor degree or above | 22（27%） | 12（29%） | 10（24%） |  |  |
| **Marital status** | | | | | |
| Unmarried | 10（12%） | 5（12%） | 5（12%） | 0 ^b^ | 1 |
| Married/divorced | 72（88%） | 36（88%） | 36（88%） |  |  |
| **Have children** | | | | | |
| No | 16（20%） | 10（24%） | 6（15%） | 1.242 ^b^ | .27 |
| Yes | 66（80%） | 31（76%） | 35（85%） |  |  |
| **Monthly household income (yuan)** | | | | | |
| ≤5000 | 42（51%） | 21（51%） | 21（51%） | 0.476 ^b^ | .79 |
| 5001-10000 | 28（34%） | 15（37%） | 13（32%） |  |  |
| ＞10000 | 12（15%） | 5（12%） | 7（17%） |  |  |
| **History of chronic pain or illness** | | |  |  |  |
| No | 71（87%） | 37（90%） | 34（83%） | 0.945 ^b^ | .33 |
| Yes | 11（13%） | 4（10%） | 7（17%） |  |  |
| **Perception of health status** | | | | | |
| Very poor/Poor/Fair | 16（20%） | 9（22%） | 7（17%） | 0.311 ^b^ | .58 |
| Good/Very good | 66（80%） | 32（78%） | 34（83%） |  |  |
| **Perception of cancer risk** | | | | | |
| Very low/Low | 43（52%） | 20（49%） | 23（56%） | 1.58 ^b^ | .45 |
| Equal to others | 27（33%） | 13（32%） | 14（34%） |  |  |
| Very high/High | 12（15%） | 8（19%） | 4（10%） |  |  |
| **Relationship with the patient** | | | | | |
| Parents/children/brothers/sisters | 47（57%） | 26（63%） | 21（51%） | 1.246 ^b^ | .26 |
| Spouse | 35（43%） | 15（37%） | 20（49%） |  |  |

**Appendix 11 (*Cont.*).**

| **Variable** | **All (n=82)** | **Intervention (n=41)** | **Control (n=41)** | ***t/χ^2^*** | ***P*** |
| --- | --- | --- | --- | --- | --- |
| **Diagnosis of the patient's disease** |  |  |  |  |  |
| Rectal cancer | 39（48%） | 24（58%） | 15（37%） | 3.961 ^b^ | .047 |
| Colon cancer | 43（52%） | 17（42%） | 26（63%） |  |  |
| **Social medical insurance of patients** | | | | | |
| No/ Urban residents/New rural cooperative medical insurance | 42（51%） | 22（54%） | 20（49%） | 0.195 ^b^ | .66 |
| Provincial/Municipal medical insurance | 40（49%） | 19（46%） | 21（51%） |  |  |
| **Commercial medical insurance of patients** | | | | | |
| No | 45（55%） | 24（59%） | 21（51%） | 0.443 ^b^ | .51 |
| Yes | 37（45%） | 17（41%） | 20（49%） |  |  |
| **Cancer-related financial distress of patients** | | | | | |
| No | 55（67%） | 26（63%） | 29（71%） | 0.497 ^b^ | .48 |
| Yes | 27（33%） | 15（37%） | 12（29%） |  |  |
| **Disease characteristics and family history of patients meet the revised Bethesda criteria** | | | | | |
| No | 56（68%） | 29（71%） | 27（66%） | 0.225 ^b^ | .64 |
| Yes | 26（32%） | 12（29%） | 14（34%） |  |  |
| **The patient's age at diagnosis of colorectal cancer** | | | | | |
| ≥50 | 66（80%） | 34（83%） | 32（78%） | 0.311 ^b^ | .58 |
| ＜50 | 16（20%） | 7（17%） | 9（22%） |  |  |
| **Number of colon/rectal tumors in the patient** | | | | | |
| 1 | 78（95%） | 40（98%） | 38（93%） | 0.263 ^b^ | .61 |
| ≥2 | 4（5%） | 1（2%） | 3（7%） |  |  |
| **The patient has other primary tumors outside the colorectum** | | | | | |
| No | 76（93%） | 38（93%） | 38（93%） | 0 ^b^ | 1 |
| Yes | 6（7%） | 3（7%） | 3（7%） |  |  |
| **The first-degree relatives of the patients had cancer and the age at diagnosis was less than 50 years** | | | | | |
| No | 79（96%） | 40（98%） | 39（95%） | 0 ^b^ | 1 |
| Yes | 3（4%） | 1（2%） | 2（5%） |  |  |
| **≥2 first - or second-degree relatives with colorectal cancer** | | | | | |
| No | 80（98%） | 41（100%） | 39（95%） | 0.513 ^b^ | .47 |
| Yes | 2（2%） | 0（0%） | 2（5%） |  |  |

^a^ indicates *t* values, ^b^ indicates ***χ^2^*** values.
