# Supplementary material for: A WeChat-Based Decision Aid Intervention to Promote Informed Decision-Making for Family Members Regarding the Genetic Testing of Patients With Colorectal Cancer: Randomized Controlled Trial
Source: J Med Internet Res. 2025 Apr 21;27:e60681. doi: 10.2196/60681 (PMC12053134; doi:10.2196/60681)
Supplement: Multimedia Appendix 8 [file jmir_v27i1e60681_app8.docx]

**Appendix 12 Baseline study outcomes and differences between groups.**

| **Variable** | **All (n=82)** | **Intervention (n=41)** | **Control (n=41)** | ***t/χ^2^/U*** | ***P*** |
| --- | --- | --- | --- | --- | --- |
| **Knowledge** | 7（5, 10） | 7.71±3.51 | 7.22±3.60 | 0.622 ^a^ | .54 |
| **Decision conflicts** | 49.43±17.40 | 47.88±16.12 | 51.01±18.66 | ﹣0.812 ^a^ | .42 |
| **Decision self-efficacy** | 34.09（19.32, 63.64） | 41.63±24.55 | 29.55（6.82，62.5） | ﹣0.785 ^c^ | .43 |
| **PCS** | 52.01（45.86, 55.5） | 51.67（46.19, 55.9） | 52.11（45.78, 54.25） | ﹣0.654 ^c^ | .51 |
| **MCS** | 51.99（43.11, 55.96） | 52.01（42.28, 56.07） | 51.05（44.2, 55.96） | ﹣0.376 ^c^ | .71 |
| **Anxiety** |  |  |  |  |  |
| No | 65（79%） | 32（78%） | 33（81%） | 1.928 ^b^ | .75 |
| Mild | 12（15%） | 6（15%） | 6（15%） |  |  |
| Moderate | 4（5%） | 3（7%） | 1（2%） |  |  |
| Severe | 1（1%） | 0（0%） | 1（2%） |  |  |
| **Depression** | | | | | |
| No | 69（84%） | 32（78%） | 37（90%） | 2.788 ^b^ | .27 |
| Mild | 11（13%） | 8（20%） | 3（7%） |  |  |
| Moderate | 2（2%） | 1（2%） | 1（2%） |  |  |
| Severe | 0（0%） | 0（0%） | 0（0%） |  |  |
| **Colorectal cancer screening in 5 years** | | | | | |
| No | 72（88%） | 33（80%） | 36（88%） | 0.823 ^b^ | .36 |
| Yes | 10（12%） | 8（20%） | 5（12%） |  |  |
| **Smoking history** | | | | | |
| No | 57（70%） | 29（71%） | 28（68%） | 0.058 ^b^ | .81 |
| Yes | 25（30%） | 12（29%） | 13（32%） |  |  |
| **Now smoking or not** | | | | | |
| No | 61（74%） | 30（73%） | 31（76%） | 0.064 ^b^ | .80 |
| Yes | 21（26%） | 11（27%） | 10（24%） |  |  |
| **Tobacco smoking (a day)** | | | | | |
| 0 | 61（74%） | 30（73%） | 31（76%） | 2.414 ^b^ | .61 |
| 1-10 | 13（16%） | 7（17%） | 6（15%） |  |  |
| 11-20 | 6（7%） | 4（10%） | 2（5%） |  |  |
| ＞20 | 2（2%） | 0（0%） | 2（5%） |  |  |
| **Drinking history** | | | | | |
| No | 27（33%） | 14（34%） | 13（32%） | 0.055 ^b^ | .81 |
| Yes | 55（67%） | 27（66%） | 28（68%） |  |  |

**Appendix 12 (*Cont.*).**

| **Variable** | **All (n=82)** | **Intervention (n=41)** | **Control (n=41)** | ***t/χ^2^/U*** | ***P*** |
| --- | --- | --- | --- | --- | --- |
| **Now drinking or not** | | |  |  |  |
| No | 33（40%） | 17（42%） | 16（39%） | 0.051 ^b^ | .82 |
| Yes | 49（60%） | 24（58%） | 25（61%） |  |  |
| **Alcohol consumption＜3 times/month** | | |  |  |  |
| No | 16（20%） | 8（20%） | 8（20%） | 0 ^b^ | 1 |
| Yes | 66（80%） | 33（80%） | 33（80%） |  |  |
| **BMI（kg/m^2^）** | 24.13±3.88 | 23.69±3.86 | 24.58±3.88 | ﹣1.042 ^a^ | .30 |
| **Waist circumference meets the recommended criteria** | | |  |  |  |
| No | 30（37%） | 14（34%） | 16（39%） | 0.21 ^b^ | .65 |
| Yes | 52（63%） | 27（66%） | 25（61%） |  |  |
| **Physical activity meets the recommended criteria** | |  |  |  |  |
| No | 33（40%） | 16（39%） | 17（42%） | 0.051 ^b^ | .82 |
| Yes | 49（60%） | 25（61%） | 24（58%） |  |  |
| **Sedentary time (h/d)** | 4（2, 6） | 3.5（2, 6） | 5（2, 6） | ﹣0.65 ^c^ | .51 |
| **Processed and red meat intake <4 times/wk** | | | | | |
| No | 32（39%） | 15（37%） | 17（42%） | 0.205 ^b^ | .65 |
| Yes | 50（61%） | 26（63%） | 24（58%） |  |  |
| **Vegetable and fruit intake >5 servings/d, 80g/serving** | | | | | |
| No | 69（84%） | 33（80%） | 36（88%） | 0.823 ^b^ | .36 |
| Yes | 13（16%） | 8（20%） | 5（12%） |  |  |
| **Healthy lifestyle scores** |  |  |  |  |  |
| Unhealthy | 4（5%） | 1（2%） | 3（7%） | 1.03 ^b^ | .73 |
| Intermediate | 19（23%） | 10（24%） | 9（22%） |  |  |
| Healthy | 59（72%） | 30（73%） | 29（71%） |  |  |
| *Note.* PCS: physical component summary; MCS: mental component summary; BMI: body mass index. ^a^ indicates *t* values, ^b^ indicates ***χ^2^*** values, ^c^ indicates *U* values. | | | | | |
