# Supplementary material for: A WeChat-Based Decision Aid Intervention to Promote Informed Decision-Making for Family Members Regarding the Genetic Testing of Patients With Colorectal Cancer: Randomized Controlled Trial
Source: J Med Internet Res. 2025 Apr 21;27:e60681. doi: 10.2196/60681 (PMC12053134; doi:10.2196/60681)
Supplement: Multimedia Appendix 9 [file jmir_v27i1e60681_app9.docx]

**Appendix 13 Baseline characteristics differences between participants who completed the study and those who withdrew.**

| **Variable** | **All (n=82)** | **Completed (n=69)** | **Withdrew (n=13)** | ***t/******χ^2^*** | ***P*** |
| --- | --- | --- | --- | --- | --- |
| **Age** | 42.39±10.91 | 42.94±11.22 | 39.46±8.86 | ﹣0.056^a^ | .29 |
| **Gender** |  |  |  |  |  |
| Male | 30（37%） | 24（35%） | 6（46%） | 0.218^b^ | .64 |
| Female | 52（63%） | 45（65%） | 7（54%） |  |  |
| **Education level** |  |  |  |  |  |
| High school and below | 35（43%） | 30（44%） | 5（39%） | 3.129 ^b^ | .22 |
| Secondary technical school/College degree | 25（30%） | 23（33%） | 2（15%） |  |  |
| Bachelor degree or above | 22（27%） | 16（23%） | 6（46%） |  |  |
| **Marital status** | | | | | |
| Unmarried | 10（12%） | 7（10%） | 3（23%） | 0.714 ^b^ | .40 |
| Married/divorced | 72（88%） | 62（90%） | 10（77%） |  |  |
| **Have children** | | | | | |
| No | 16（20%） | 12（17%） | 4（31%） | 0.54 ^b^ | .46 |
| Yes | 66（80%） | 57（83%） | 9（69%） |  |  |
| **Monthly household income (yuan)** | | | | | |
| ≤5000 | 42（51%） | 36（52%） | 6（46%） | 0.352 ^b^ | .92 |
| 5001-10000 | 28（34%） | 23（33%） | 5（39%） |  |  |
| ＞10000 | 12（15%） | 10（15%） | 2（15%） |  |  |
| **History of chronic pain or illness** | | |  |  |  |
| No | 71（87%） | 58（84%） | 13（100%） | 1.218 ^b^ | .27 |
| Yes | 11（13%） | 11（16%） | 0（0%） |  |  |
| **Perception of health status** | | | | | |
| Very poor/Poor/Fair | 16（20%） | 14（20%） | 2（15%） | 0.001 ^b^ | .98 |
| Good/Very good | 66（80%） | 55（80%） | 11（85%） |  |  |
| **Perception of cancer risk** | | | | | |
| Very low/Low | 43（52%） | 36（52%） | 7（54%） | 2.908 ^b^ | .24 |
| Equal to others | 27（33%） | 21（30%） | 6（46%） |  |  |
| Very high/High | 12（15%） | 12（17%） | 0（0%） |  |  |
| **Relationship with the patient** | | | | | |
| Parents/children/brothers/sisters | 47（57%） | 39（57%） | 8（62%） | 0.113 ^b^ | .74 |
| Spouse | 35（43%） | 30（43%） | 5（38%） |  |  |

**Appendix 13 (*Cont.*).**

| **Variable** | **All (n=82)** | **Completed (n=69)** | **Withdrew (n=13)** | ***t/χ^2^*** | ***P*** |
| --- | --- | --- | --- | --- | --- |
| **Diagnosis of the patient's disease** |  |  |  |  |  |
| Rectal cancer | 39（48%） | 35（51%） | 4（31%） | 1.747 ^b^ | .19 |
| Colon cancer | 43（52%） | 34（49%） | 9（69%） |  |  |
| **Social medical insurance of patients** | | | | | |
| No/ Urban residents/New rural cooperative medical insurance | 42（51%） | 36（52%） | 6（46%） | 0.159 ^b^ | .69 |
| Provincial/Municipal medical insurance | 40（49%） | 33（48%） | 7（54%） |  |  |
| **Commercial medical insurance of patients** | | | | | |
| No | 45（55%） | 37（54%） | 8（62%） | 0.277 ^b^ | .60 |
| Yes | 37（45%） | 32（46%） | 5（38%） |  |  |
| **Cancer-related financial distress of patients** | | | | | |
| No | 55（67%） | 44（64%） | 11（85%） | 1.312 ^b^ | .25 |
| Yes | 27（33%） | 25（36%） | 2（15%） |  |  |
| **Disease characteristics and family history of patients meet the revised Bethesda criteria** | | | | | |
| No | 56（68%） | 46（67%） | 10（77%） | 0.163 ^b^ | .69 |
| Yes | 26（32%） | 23（33%） | 3（23%） |  |  |
| **The patient's age at diagnosis of colorectal cancer** | | | | | |
| ≥50 | 66（80%） | 55（80%） | 11（85%） | 0.001 ^b^ | .98 |
| ＜50 | 16（20%） | 14（20%） | 2（15%） |  |  |
| **Number of colon/rectal tumors in the patient** | | | | | |
| 1 | 78（95%） | 65（94%） | 13（100%） | -- ^b^ | 1 |
| ≥2 | 4（5%） | 4（6%） | 0（0%） |  |  |
| **The patient has other primary tumors outside the colorectum** | | | | | |
| No | 76（93%） | 64（93%） | 12（92%） | -- ^b^ | 1 |
| Yes | 6（7%） | 5（7%） | 1（8%） |  |  |
| **The first-degree relatives of the patients had cancer and the age at diagnosis was less than 50 years** | | | | | |
| No | 79（96%） | 66（96%） | 13（100%） | -- ^b^ | 1 |
| Yes | 3（4%） | 3（4%） | 0（0%） |  |  |
| **≥2 first - or second-degree relatives with colorectal cancer** | | | | | |
| No | 80（98%） | 67（97%） | 13（100%） | -- ^b^ | 1 |
| Yes | 2（2%） | 2（3%） | 0（0%） |  |  |

**Appendix 13 (Cont.).**

| **Variable** | **All (n=82)** | **Completed (n=69)** | **Withdrew (n=13)** | ***t/χ^2^/U*** | ***P*** |
| --- | --- | --- | --- | --- | --- |
| **Knowledge** | 7（5, 10） | 7.71±3.59 | 6.15±3.08 | 1.465 ^a^ | .15 |
| **Decision conflicts** | 49.43±17.40 | 49.07±18.33 | 51.32±11.60 | ﹣0.426^a^ | .67 |
| **Decision self-efficacy** | 34.09（19.32, 63.64） | 40.94±27.70 | 35.84±27.65 | 0.609^a^ | .54 |
| **PCS** | 52.01（45.86, 55.5） | 49.45±7.69 | 52.21±6.89 | ﹣1.206 ^a^ | .23 |
| **MCS** | 51.99（43.11, 55.96） | 48.35±10.41 | 50.93±6.98 | ﹣0.858 ^a^ | .39 |
| **Anxiety** |  |  |  |  |  |
| No | 65（79%） | 54（78%） | 11（85%） | 1.571 ^b^ | .65 |
| Mild | 12（15%） | 11（16%） | 1（8%） |  |  |
| Moderate | 4（5%） | 3（4%） | 1（7%） |  |  |
| Severe | 1（1%） | 1（1%） | 0（0%） |  |  |
| **Depression** | | | | | |
| No | 69（84%） | 58（84%） | 11（85%） | 0.419 ^b^ | 1 |
| Mild | 11（13%） | 9（13%） | 2（15%） |  |  |
| Moderate | 2（2%） | 2（3%） | 0（0%） |  |  |
| Severe | 0（0%） | 0（0%） | 0（0%） |  |  |
| **Colorectal cancer screening in 5 years** | | | | | |
| No | 72（88%） | 59（86%） | 13（100%） | 1.006 ^b^ | .32 |
| Yes | 10（12%） | 10（14%） | 0（0%） |  |  |
| **Smoking history** | | | | | |
| No | 57（70%） | 49（71%） | 8（62%） | 0.124 ^b^ | .73 |
| Yes | 25（30%） | 20（29%） | 5（38%） |  |  |
| **Now smoking or not** | | | | | |
| No | 61（74%） | 53（77%） | 8（62%） | 0.658 ^b^ | .42 |
| Yes | 21（26%） | 16（23%） | 5（38%） |  |  |
| **Tobacco smoking (a day)** | | | | | |
| 0 | 61（74%） | 53（77%） | 8（62%） | 2.77^b^ | .43 |
| 1-10 | 13（16%） | 10（15%） | 3（23%） |  |  |
| 11-20 | 6（7%） | 5（7%） | 2（15%） |  |  |
| ＞20 | 2（2%） | 1（1%） | 0（0%） |  |  |
| **Drinking history** | | | | | |
| No | 27（33%） | 20（29%） | 7（54%） | 2.039 ^b^ | .15 |
| Yes | 55（67%） | 49（71%） | 6（46%） |  |  |

**Appendix 13 (*Cont.*).**

| **Variable** | **All (n=82)** | **Completed (n=69)** | **Withdrew (n=13)** | ***t/χ^2^/U*** | ***P*** |
| --- | --- | --- | --- | --- | --- |
| **Now drinking or not** | | |  |  |  |
| No | 33（40%） | 25（36%） | 8（62%） | 2.913 ^b^ | .09 |
| Yes | 49（60%） | 44（64%） | 5（38%） |  |  |
| **Alcohol consumption＜3 times/month** | | |  |  |  |
| No | 16（20%） | 15（22%） | 1（8%） | 0.625 ^b^ | .43 |
| Yes | 66（80%） | 54（78%） | 12（92%） |  |  |
| **BMI（kg/m^2^）** | 24.13±3.88 | 24.29±4.03 | 23.32±2.89 | 0.822 ^a^ | .41 |
| **Waist circumference meets the recommended criteria** | | |  |  |  |
| No | 30（37%） | 27（39%） | 3（23%） | 0.622^b^ | .43 |
| Yes | 52（63%） | 42（61%） | 10（77%） |  |  |
| **Physical activity meets the recommended criteria** | |  |  |  |  |
| No | 33（40%） | 26（38%） | 7（54%） | 1.189 ^b^ | .28 |
| Yes | 49（60%） | 43（62%） | 6（46%） |  |  |
| **Sedentary time (h/d)** | 4（2, 6） | 4（2, 6） | 6（2, 8） | -- ^c^ | .33 |
| **Processed and red meat intake <4 times/wk** | | | | | |
| No | 32（39%） | 25（36%） | 7（54%） | 1.426 ^b^ | .23 |
| Yes | 50（61%） | 44（64%） | 6（46%） |  |  |
| **Vegetable and fruit intake >5 servings/d, 80g/serving** | | | | | |
| No | 69（84%） | 58（84%） | 11（85%） | 0 ^b^ | 1 |
| Yes | 13（16%） | 11（16%） | 2（15%） |  |  |
| **Healthy lifestyle scores** |  |  |  |  |  |
| Unhealthy | 4（5%） | 2（3%） | 2（15%） | 7.516 ^b^ | .02 |
| Intermediate | 19（23%） | 19（27%） | 0（0%） |  |  |
| Healthy | 59（72%） | 48（70%） | 11（85%） |  |  |
| *Note.* PCS: physical component summary; MCS: mental component summary; BMI: body mass index. ^a^ indicates *t* values, ^b^ indicates ***χ^2^*** values, ^c^ indicates *U* values. | | | | | |
