# Supplementary material for: A WeChat-Based Decision Aid Intervention to Promote Informed Decision-Making for Family Members Regarding the Genetic Testing of Patients With Colorectal Cancer: Randomized Controlled Trial
Source: J Med Internet Res. 2025 Apr 21;27:e60681. doi: 10.2196/60681 (PMC12053134; doi:10.2196/60681)
Supplement: Multimedia Appendix 10 [file jmir_v27i1e60681_app10.docx]

**Appendix 4 Participants’ adherence of intervention.**

|  | **T1** | | **T2** | |
| --- | --- | --- | --- | --- |
|  | **Frequency** | **Percentage (%)** | **Frequency** | **Percentage (%)** |
| **Lost to follow-up** | 7 | 17 | 7 | 17 |
| **Have not read** | 7 | 17 | 4 | 10 |
| **Read a part of DA** | 14 | 34 | 7 | 17 |
| **A brief reading for all DA** | 5 | 12 | 7 | 17 |
| **Read carefully for all DA** | 8 | 20 | 16 | 39 |

*Note.* DA: decision aid.
