# Supplementary material for: A WeChat-Based Decision Aid Intervention to Promote Informed Decision-Making for Family Members Regarding the Genetic Testing of Patients With Colorectal Cancer: Randomized Controlled Trial
Source: J Med Internet Res. 2025 Apr 21;27:e60681. doi: 10.2196/60681 (PMC12053134; doi:10.2196/60681)
Supplement: Multimedia Appendix 11 [file jmir_v27i1e60681_app11.docx]

**Appendix 5 Outcomes of two groups at T1 and T2.**

| **Variable** | | **Intervention (n=41)** | **Control (n=41)** |
| --- | --- | --- | --- |
|  |  | **n (%)/Mean±SD/Median (IQRs)** | **n (%)/Mean±SD/Median (IQRs)** |
| **Knowledge** | | | |
| T1 | | 11（7,14） | 7.37±3.75 |
| T2 | | 12（9,14） | 7.37±3.75 |
| **Decision conflicts** | | | |
| T1 | | 32.8（17.2,47.7） | 48.32±19.35 |
| T2 | | 23.4（12.5,39.8） | 48.32±19.35 |
| **Decision self-efficacy** | | | |
| T1 | | 63.6（35.2,80.7） | 31.8（6.8,62.5） |
| T2 | | 75.0,47.7,81.8） | 31.8（6.8,62.5） |
| **PCS** | | | |
| T1 | | 52.4（46.2,55.9） | 52.1（45.6,54.3） |
| T2 | | 52.1（45.5,56.0） | 52.1（45.6,54.3） |
| **MCS** | | | |
| T1 | | 52.0（41.4,56.1） | 51.0（44.2,56.0） |
| T2 | | 49.1（41.4,56.1） | 51.0（44.2,56.0） |
| **Anxiety** | | | |
| T1 | No | 33（81%） | 33（81%） |
|  | Mild | 5（12%） | 6（15%） |
|  | Moderate | 3（7%） | 1（2%） |
|  | Severe | 0（0%） | 1（2%） |
| T2 | No | 33（81%） | 33（81%） |
|  | Mild | 5（12%） | 6（15%） |
|  | Moderate | 3（7%） | 1（2%） |
|  | Severe | 0（0%） | 1（2%） |
| **Depression** | | | |
| T1 | No | 31（76%） | 37（90%） |
|  | Mild | 9（22%） | 3（7%） |
|  | Moderate | 1（2%） | 1（2%） |
|  | Severe | 0（0%） | 0（0%） |
| T2 | No | 31（76%） | 37（90%） |
|  | Mild | 9（22%） | 3（7%） |
|  | Moderate | 1（2%） | 1（2%） |
|  | Severe | 0（0%） | 0（0%） |

**Appendix 5** **(*Cont.*).**

| **Variable** | | **Intervention（n=41）** | **Control（n=41）** |
| --- | --- | --- | --- |
|  |  | **n (%)/Mean±SD/Median (IQRs)** | **n (%)/Mean±SD/Median (IQRs)** |
| **CRC screening in 5 years** | | | |
| T1 | No | 29（71%） | 35（85%） |
|  | Yes | 12（29%） | 6（15%） |
| T2 | No | 25（61%） | 33（80%） |
|  | Yes | 16（39%） | 8（20%） |
| **Alcohol consumption＜3 times/month** | | | |
| T1 | No | 8（20%） | 7（17%） |
|  | Yes | 33（80%） | 34（83%） |
| T2 | No | 7（17%） | 8（20%） |
|  | Yes | 34（83%） | 33（80%） |
| **Tobacco smoking (a day)** | | | |
| T1 | 0 | 30（73%） | 32（78%） |
|  | 1-10 | 8（20%） | 5（12%） |
|  | 11-20 | 3（7%） | 2（5%） |
|  | ＞20 | 0（0%） | 2（5%） |
| T2 | 0 | 30（73%） | 32（78%） |
|  | 1-10 | 8（20%） | 5（12%） |
|  | 11-20 | 3（7%） | 2（5%） |
|  | ＞20 | 0（0%） | 2（5%） |
| **BMI (kg/m^2^)** | | | |
| T1 | | 23.7±3.8 | 24.6±3.9 |
| T2 | | 23.7±3.8 | 24.6±3.9 |
| **Waist circumference meets the recommended criteria** | | | |
| T1 | No | 15（37%） | 18（44%） |
|  | Yes | 26（63%） | 23（56%） |
| T2 | No | 15（37%） | 18（44%） |
|  | Yes | 26（63%） | 23（56%） |
| **Physical activity meets the recommended criteria** | | | |
| T1 | No | 15（37%） | 16（39%） |
|  | Yes | 26（63%） | 25（61%） |
| T2 | No | 15（37%） | 16（39%） |
|  | Yes | 26（63%） | 25（61%） |
| **Sedentary time (h/d)** | | | |
| T1 | | 3.5（2,5.75） | 4（2,6） |
| T2 | | 3.5（2,5.75） | 4（2,6） |

**Appendix 5 (*Cont.*).**

| **Variable** | | **Intervention（n=41）** | **Control（n=41）** |
| --- | --- | --- | --- |
|  |  | **n (%)/Mean±SD/Median (IQRs)** | **n (%)/Mean±SD/Median (IQRs)** |
| **Processed and red meat intake <4 times/wk** | | | |
| T1 | No | 15（37%） | 17（42%） |
|  | Yes | 26（63%） | 24（58%） |
| T2 | No | 14（34%） | 17（42%） |
|  | Yes | 27（66%） | 24（58%） |
| **Vegetable and fruit intake >5 servings/d, 80g/serving** | | | |
| T1 | No | 30（73%） | 33（80%） |
|  | Yes | 11（27%） | 8（20%） |
| T2 | No | 30（73%） | 33（80%） |
|  | Yes | 11（27%） | 8（20%） |
| **Healthy lifestyle scores** | | | |
| T1 | unhealthy | 1（2%） | 3（7%） |
|  | intermediate | 8（20%） | 8（20%） |
|  | healthy | 32（78%） | 30（73%） |
| T2 | unhealthy | 1（2%） | 3（7%） |
|  | intermediate | 8（20%） | 10（24%） |
|  | healthy | 32（78%） | 28（68%） |

*Note.* SD: Standard Deviation; IQR: Inter Quartile Range; PCS: physical component summary;

MCS: mental component summary; CRC: colorectal cancer; BMI: body mass index.
