# Supplementary material for: A WeChat-Based Decision Aid Intervention to Promote Informed Decision-Making for Family Members Regarding the Genetic Testing of Patients With Colorectal Cancer: Randomized Controlled Trial
Source: J Med Internet Res. 2025 Apr 21;27:e60681. doi: 10.2196/60681 (PMC12053134; doi:10.2196/60681)
Supplement: Multimedia Appendix 12 [file jmir_v27i1e60681_app12.docx]

**Appendix 6 Comparison preparation for decision making, genetic counseling and genetic testing between T1 and T2.**

| **Variable** | | **Intervention（n=41）** | | | **Control（n=41）** | | |
| --- | --- | --- | --- | --- | --- | --- | --- |
|  |  | **n(%)/Mean±SD/Median (IQRs)** | **χ^2^** | ***P*** | **n(%)/Mean±SD/Median (IQRs)** | **χ^2^** | ***P*** |
| **Preparation for Decision Making** | | | | | | | |
| T1 | ＜60 | 25（61%） |  |  | — |  |  |
|  | ≥60 | 16（39%） |  |  | — |  |  |
| T2 | ＜60 | 21（51%） | 2.25 | .13 | — | — | — |
|  | ≥60 | 20（49%） |  |  | — |  |  |
| **Genetic counseling** | | | | | | | |
| T1 | No | 40（98%） |  |  | 39（95%） |  |  |
|  | Yes | 1（2%） |  |  | 2（5%） |  |  |
| T2 | No | 39（95%） | 0 | 1 | 39（95%） | 0 | 1 |
|  | Yes | 2（5%） |  |  | 2（5%） |  |  |
| **Genetic testing (patients)** | | | | | | | |
| T1 | No | 40（98%） |  |  | 40（98%） |  |  |
|  | Yes | 1（2%） |  |  | 1（2%） |  |  |
| T2 | No | 39（95%） | 0 | 1 | 40（98%） | 0 | 1 |
|  | Yes | 2（5%） |  |  | 1（2%） |  |  |

*Note.* SD: Standard Deviation; IQR: Inter Quartile Range.
