# Supplementary material for: A WeChat-Based Decision Aid Intervention to Promote Informed Decision-Making for Family Members Regarding the Genetic Testing of Patients With Colorectal Cancer: Randomized Controlled Trial
Source: J Med Internet Res. 2025 Apr 21;27:e60681. doi: 10.2196/60681 (PMC12053134; doi:10.2196/60681)
Supplement: Multimedia Appendix 13 [file jmir_v27i1e60681_app13.docx]

**Appendix 14 Generalized estimating equation models of the comparison of study outcomes between the intervention and control groups.**

| **Variables** | **Time effect** | | **Group effect** | | **Group*Time effect** | | **Effect size** |
| --- | --- | --- | --- | --- | --- | --- | --- |
|  | ***β*(95%CI)** | ***P*** | ***β*(95%CI)** | ***P*** | ***β*(95%CI)** | ***P*** |  |
| **Knowledge** | | | 2.172(0.650, 3.694) | .005 |  |  |  |
| **T1** | 1.171(0.668, 1.673) | ＜.001 |  |  | 2.049(1.147, 2.950) | ＜.001 | 0.63^a^ |
| **T2** | 1.805(1.172, 2.438) | ＜.001 |  |  | 3.317(2.275, 4.359) | ＜.001 | 0.96^a^ |
| **Decision Conflicts** | | | ﹣12.170(﹣19.202, ﹣5.137) | .001 |  |  |  |
| **T1** | ﹣8.512(﹣11.648, ﹣5.376) | ＜.001 |  |  | ﹣11.660(﹣17.402, ﹣5.918) | ＜.001 | 0.77^a^ |
| **T2** | ﹣11.465(﹣15.068, ﹣7.862) | ＜.001 |  |  | ﹣17.587(﹣23.689, ﹣11.445) | ＜.001 | 1.13^a^ |
| **Decision Self-Efficacy** | | | 14.407(3.014, 25.801) | .01 |  |  |  |
| **T1** | 9.344(5.601, 13.087) | ＜.001 |  |  | 15.353(8.645, 22.061) | ＜.001 | 0.64^a^ |
| **T2** | 12.863(8.687, 16.985) | ＜.001 |  |  | 22.337(15.593, 29.082) | ＜.001 | 0.91^a^ |
| **PCS** | | | 1.323(﹣2.090, 4.736) | .45 |  |  |  |
| **T1** | 0.414(﹣0.549, 1.377) | .40 |  |  | 0.967(﹣0.948, 2.881) | .32 | 0.23^a^ |
| **T2** | 0.162(﹣0.961, 1.285) | .78 |  |  | 0.463(﹣1.780, 2.706) | .69 | 0.17^a^ |
| **MCS** | | | ﹣0.921(﹣5.122, 3.280) | .67 |  |  |  |
| **T1** | ﹣0.035(﹣0.440, 0.371) | .87 |  |  | ﹣0.102(﹣0.913, 0.709) | .81 | 0.09^a^ |
| **T2** | ﹣0.132(﹣0.601, 0.337) | .58 |  |  | ﹣0.296(﹣1.231, 0.638) | .53 | 0.11^a^ |
| **Anxiety** | | | 0.262(﹣0.822, 1.347) | .64 |  |  |  |
| **T1** | ﹣0.077(﹣0.225, 0.070) | .30 |  |  | ﹣0.073(﹣0.380, 0.234) | .64 | 0.16^b^ |
| **T2** | ﹣0.069(﹣0.204, 0.066) | .32 |  |  | ﹣0.127(﹣0.386, 0.131) | .34 | 0.16^b^ |
| **Depression** | | | 1.497(﹣0.139, 3.133) | .07 |  |  |  |
| **T1** | 0.098(﹣0.110, 0.307) | .36 |  |  | 0.171(﹣0.130, 0.473) | .27 | 0.21^b^ |
| **T2** | 0.098(﹣0.110, 0.307) | .36 |  |  | 0.151(﹣0.145, 0.448) | .32 | 0.21^b^ |
| **CRC screening in 5 years** | | | 0.962(﹣0.015, 1.939) | .05 |  |  |  |
| **T1** | 0.726(0.255, 1.197) | .003 |  |  | ﹣0.672(﹣1.967, 0.623) | .31 | 0.18^c^ |
| **T2** | 1.123(0.581, 1.665) | ＜.001 |  |  | ﹣0.583(﹣1.997, 0.832) | .42 | 0.21^c^ |
| **Tobacco smoking** | | | ﹣0.073(﹣1.175, 1.028) | .90 |  |  |  |
| **T1** | ﹣0.109(﹣0.255, 0.037) | .14 |  |  | 0.258(﹣0.071, 0.587) | .13 | 0.19^b^ |
| **T2** | ﹣0.135(﹣0.296. 0.026) | .10 |  |  | 0.206(﹣0.123,0.536) | .22 | 0.19^b^ |
| **Alcohol consumption** | | | 0.109(﹣0.961, 1.180) | .84 |  |  |  |
| **T1** | 0.080(﹣0.076, 0.235) | .32 |  |  | ﹣0.163(﹣0.480, 0.153) | .31 | 0.03^c^ |
| **T2** | 0.080(﹣0.190, 0.349) | .56 |  |  | 0.163(﹣0.371, 0.698) | .55 | 0.03^c^ |
| **BMI（kg/m^2^）** | | | ﹣0.915(﹣2.559, 0.729) | .28 |  |  |  |
| **T1** | 0.011(﹣0.082, 0.104) | .81 |  |  | ﹣0.016(﹣0.202, 0.170) | .86 | 0.23^a^ |
| **T2** | ﹣0.001(﹣0.097, 0.095) | .99 |  |  | ﹣0.040(﹣0.233, 0.152) | .68 | 0.23^a^ |

**Appendix 14 (*Cont.*).**

| **Variables** | **Time effect** | | **Group effect** | | **Group*Time effect** | | **Effect size** |
| --- | --- | --- | --- | --- | --- | --- | --- |
|  | ***β*(95%CI)** | ***P*** | ***β*(95%CI)** | ***P*** | ***β*(95%CI)** | ***P*** |  |
| **Waist circumference** | | | 0.206(﹣0.679, 1.091) | .65 |  |  |  |
| **T1** | ﹣0.104(﹣0.246, 0.038) | .15 |  |  | ﹣0.005(﹣0.290, 0.280) | .97 | 0.08^c^ |
| **T2** | ﹣0.104(﹣0.246, 0.038) | .15 |  |  | ﹣0.005(﹣0.290, 0.280) | .97 | 0.08^c^ |
| **Physical activity** | |  | 0.102(﹣0.777, 0.980) | .82 |  |  |  |
| **T1** | 0.103(﹣0.038, 0.243) | .15 |  |  | 0.002(﹣0.279, 0.283) | .99 | 0.03^c^ |
| **T2** | 0.103(﹣0.038, 0.243) | .15 |  |  | 0.002(﹣0.279, 0.283) | .99 | 0.03^c^ |
| **Sedentary time (h/d)** | | | ﹣0.317(﹣1.610, 0.976) | .63 |  |  |  |
| **T1** | ﹣0.122(﹣0.207, ﹣0.037) | .005 |  |  | ﹣0.098(﹣0.267, 0.072) | .26 | 0.12^a^ |
| **T2** | ﹣0.122(﹣0.207, ﹣0.037) | .005 |  |  | ﹣0.098(﹣0.267, 0.072) | .26 | 0.12^a^ |
| **Processed and red meat intake** | | | ﹣0.190(﹣1.080, 0.701) | .68 |  |  |  |
| **T1** | ﹣3.060(﹣2.764, 8.885) | .30 |  |  | ﹣1.494(﹣1.494,﹣1.494) | ＜.001 | 0.05^c^ |
| **T2** | ﹣0.052(﹣0.152, 0.049) | .31 |  |  | ﹣0.107(﹣0.313, 0.100) | .31 | 0.08^c^ |
| **Vegetable and fruit intake** | | | 0.391(﹣0.665, 1.447) | .47 |  | |  |
| **T1** | 0.473(0.108, 0.839) | .01 |  |  | ﹣0.143(﹣0.907, 0.621) | .71 | 0.09^c^ |
| **T2** | 0.473(0.107, 0.838) | .01 |  |  | ﹣0.143(﹣0.907, 0.621) | .71 | 0.09^c^ |
| **Healthy lifestyle scores (HLSs)** | | | 0.531(﹣0.425, 1.487) | .28 |  |  |  |
| **T1** | 0.247(﹣0.024, 0.519) | .07 |  |  | 0.481(﹣0.139, 1.101) | .13 | 0.11^b^ |
| **T2** | 0.255(﹣0.223, 0.733) | .30 |  |  | 1.073(0.081, 2.064) | .03 | 0.14^b^ |

*Note.* PCS: physical component summary; MCS: mental component summary; CRC: colorectal cancer; BMI: body mass index; ^a^: Cohen’s *d*; ^b^: Cramer's V; ^c^: Phi.
