# Supplementary material for: A WeChat-Based Decision Aid Intervention to Promote Informed Decision-Making for Family Members Regarding the Genetic Testing of Patients With Colorectal Cancer: Randomized Controlled Trial
Source: J Med Internet Res. 2025 Apr 21;27:e60681. doi: 10.2196/60681 (PMC12053134; doi:10.2196/60681)
Supplement: Multimedia Appendix 14 [file jmir_v27i1e60681_app14.docx]

**Appendix 7 Satisfaction with the decision aid intervention.**

| **Variable** | | **Frequency** | **Percentage (%)** |
| --- | --- | --- | --- |
| **Overall satisfaction** | Satisfaction | 28 | 68 |
|  | Fair | 1 | 2 |
|  | Dissatisfaction | 1 | 2 |
|  | Not Applicable* | 11 | 27 |
| **Intelligibility** | Good | 22 | 54 |
|  | Fair | 6 | 15 |
|  | Poor | 2 | 5 |
|  | Not Applicable* | 11 | 27 |
| **Adequacy** | Adequate | 27 | 66 |
|  | A little too much | 3 | 7 |
|  | Not Applicable* | 11 | 27 |
| **The method of information delivery** | Satisfaction | 30 | 73 |
|  | Fair | 0 | 0 |
|  | Dissatisfaction | 0 | 0 |
|  | Not Applicable* | 11 | 27 |
| *Note.* * Not Applicable include participants who lost to follow-up and did not read at all. | | | |
